# High Visceral Adipose Tissue Density Correlates With Unfavorable Outcomes in Patients With Intermediate-Stage Hepatocellular Carcinoma Undergoing Transarterial Chemoembolization

**DOI:** 10.3389/fcell.2021.710104

**Published:** 2021-09-08

**Authors:** Qiang Li, Lei Zhang, Zhong-Heng Hou, Dong-Xu Zhao, Jian-Bin Li, Shuai Zhang, Yu Yin, Cai-Fang Ni, Tao Chen

**Affiliations:** ^1^Department of Radiology, The Affiliated People’s Hospital of Ningbo University, Ningbo, China; ^2^Department of Interventional Radiology, The First Affiliated Hospital of Soochow University, Suzhou, China; ^3^Department of General Surgery, Jiangsu Province Hospital, The First Affiliated Hospital with Nanjing Medical University, Nanjing, China

**Keywords:** body composition, hepatocellular carcinoma, computed tomography, transarterial chemoembolization, adipose tissue

## Abstract

**Objectives:** This study aimed to evaluate the association between different body composition features with prognostic outcomes of intermediate stage hepatocellular carcinoma (HCC) patients treated with transarterial chemoembolization (TACE).

**Methods:** The areas and density of skeletal muscle area (SM) and adipose tissue [subcutaneous (SAT); visceral (VAT)] were calculated on the pre-TACE CT scans. Overall survival (OS) and progression-free survival (PFS) curves were calculated using the Kaplan–Meier method and compared with log-rank test. The discrimination and performance of body composition features were measured by area under time-dependent receiver operating characteristic (ROC) curve. Univariate and multivariate Cox proportional hazard analyses were applied to identify the association between body composition parameters and outcomes.

**Results:** A significant prolonged OS and PFS was displayed by Kaplan–Meier curve analysis for HCC patients with VAT HU below −89.1 (25.1 months, 95% CI: 18.1–32.1 vs. 17.6 months, 95% CI: 16.3–18.8, *p* < 0.0001, 15.4 months, 95% CI: 10.6–20.2 vs. 6.6 months, 95% CI: 4.9–8.3, *p* < 0.0001, respectively). The 1-, 2-, 3-, and 5-year OS area under the curve (AUC) values of the VAT HU were higher than the other body composition parameters. Meanwhile, it is also found that 3-, 6-, 9-, and 12-month PFS AUC values of VAT HU were the highest among all the parameters. Univariate and multivariate Cox-regression analysis suggested a significant association between VAT density and outcomes (OS, HR: 1.015, 95% CI: 1.004–1.025, *p* = 0.005, PFS, HR: 1.026, 95% CI: 1.016–1.036, *p* < 0.0001, respectively).

**Conclusion:** The VAT density could provide prognostic prediction value and may be helpful to stratify the intermediate stage HCC patients.

## Introduction

Transarterial chemoembolization (TACE) is the standard treatment modality for patients with intermediate stage hepatocellular carcinoma (HCC) according to the widely applied Barcelona Clinic of Liver Cancer (BCLC) staging system (Marrero et al., 2018; Villanueva, 2019). However, the response rates and survival are heterogeneous, and the target patients who will benefit particularly well from TACE is still controversial (Forner et al., 2018). In brief, tumor burden, liver function, and etiology have great influence on the prognosis of BCLC B stage HCC patients. Additionally, simple predictive algorithms including 6 to 12, up to 7, HAP, BCLC sub-classifications, which are mainly based on the tumor number and size as well as Child–Pugh class, were suggested for making decisions for those patients with significant degree of disease heterogeneity (Kim et al., 2017; Lee et al., 2019; Wang et al., 2019). Nevertheless, considering that the predictive value of these parameters could not be validated in larger clinical trials, it is crucial that novel pre-treatment stratification strategies are corroborated in order to improve the overall survival (OS) for intermediate stage HCC patients.

In recent years, previous studies demonstrated that the sarcopenia, which is defined as the declination in muscle volume mass and strength was associated with poor outcomes of HCC patients (Fujiwara et al., 2015; Antonelli et al., 2018; Cruz-Jentoft and Sayer, 2019; Hamaguchi et al., 2019; Qayyum et al., 2021). However, the correlation between sarcopenia and outcomes such as tumor response and survival in intermediate stage HCC patients undergoing TACE treatment has not been largely evaluated (Marasco et al., 2020). In addition, the prognostic role of the pre-treatment skeletal muscle remains debated (Loosen et al., 2019). More recently, the increased visceral adiposity tissue (VAT) has been suggested as an independent risk factor for recurrence after resection in HCC patients (Imai et al., 2021). Nevertheless, its predictive value has not been well studied in these patients receiving TACE treatment. Except from the quantitative measurements of body composition, the mean tissue attenuation expressed in Housfield units (HU) can also offer a qualitative information as well as provide perception into the pathophysiology (Larsen et al., 2020). By contrast, although mean tissue attenuation is easily measured on the computed tomography (CT) images, limit data are shown regarding the prognostic impact of tissue density in BCLC B stage HCC patients.

This study aimed to evaluate the association of skeletal muscle and adipose tissue mass and density with prognostic outcomes of intermediate stage HCC patients treated with TACE.

## Materials and Methods

### Study Population

The current study was reviewed and approved by the Institutional Review Board of the Soochow University and was conducted in accordance with the ethical standards laid down in the Declaration of Helsinki. Consecutive treatment-naïve intermediate-stage HCC patients undergoing TACE treatment between 2008 and 2018 were screened in this retrospective cohort study. The diagnosis of HCC was based on the non-invasive criteria [computed tomography (CT) or magnetic resonance (MR) imaging] or histological assessments. The inclusion criteria were as follows: (i) age >18 years, (ii) no prior HCC-related treatment (resection, ablation, systemic, and radiation therapy), (iii) Eastern Cooperative Oncology Group (ECOG) score 0 or 1, (iv) available CT scans at baseline. Among 256 patients, 209 patients met the inclusion criteria. Patients with decompensated liver function (*n* = 12), severe renal dysfunction (*n* = 3), and malignancy other than HCC (*n* = 2) were excluded. Finally, 192 HCC patients were included in this study. Patient and imaging data were anonymized and extracted from the electronic patient record system.

### Transarterial Chemoembolization Treatment

Briefly, TACE was performed as selectively as possible through the segmental or subsegmental hepatic arteries according to the extent of tumor burden and patient’s hepatic reserve. To identify the location and all of the feeding vessels of tumor, a thorough angiography was performed. An emulsion of 5–20 ml of iodized oil (Lipiodol; Guerbet Laboratories, Roissy, France) and 20–40 mg epirubicin hydrochloride (Shenzhen Main Luck Pharmaceutical Inc, Shenzhen, China) was infused into the feeding arteries using a 2.7F microcatheter (Renegade; Boston Scientific, Marlborough, Massachusetts; or Progreat; Terumo, Japan). This was followed by particle embolization with Gelfoam (Ailikang Inc, Hangzhou, China) until stasis in a second- or third-order branch was achieved. Repeated TACE treatments were conducted when vital tumor tissue was observed on the contrast-enhanced CT or MRI at every 6–8 weeks. All TACE procedures were performed by one of five interventional radiologists with more than 8 years of experience.

### Computed Tomography Scan Analysis

All CT scans including non-contrast scan and contrast-enhanced triple phases were performed with the Siemens SOMATOM Sensation 64 CT scanner (Erlangen, Germany) within 7 days before the initial TACE treatment. For standardized analysis of each patient, a cross-sectional enhanced CT images at the third lumbar vertebra (L3) was selected. The areas of skeletal muscle area (SM) and adipose tissue [subcutaneous, (SAT); visceral (VAT)] were measured by using the Slice-O-Matic software (version 5.0; Tomovision, Montreal, Canada), and the calculation was based on the Hounsfield units (HU) thresholds (−29 to 150 HU for SM, −190 to −30 for SAT and −150 to −50 for VAT) (von Hessen et al., 2021). All of the three variables were normalized for the height in m^2^ and expressed as indexes (cm^2^/m^2^). The skeletal muscles at L3 consisted of the psoas major, the erector spinae, the quadratus lumborum, the rectus abdominis, the transversus abdominis, the internal oblique, and the external oblique. Additionally, the density of each variable was calculated in HU. For the measurements of the variables, two observers with more than 5 years of experience in abdominal radiology independently measured the CT images from 30 randomly selected patients and compared the results, and the inter-observer agreement was 97.0%.

### Outcomes, Assessments, and Follow-Up

All HCC patients received routine blood tests and biochemistry tests before the initial TACE treatment, at 1 month after each TACE and, thereafter, every 8–12 weeks. Tumor response was evaluated with contrast-enhanced CT or MRI after first TACE treatment, 4 weeks after each TACE treatment bias according to modified Response Evaluation Criteria in Solid Tumors (mRECIST) criteria. Through the PACS system (NEUSOFTPACS/RIS, Shengyang Neusoft Co., Ltd, China), assessment of tumor response was performed on the target lesion by two radiologists with more than 5 years of experience in diagnostic radiology and divided into two groups (responder, complete response, and partial response; non-responder, stable disease, and progressive disease). For the survival follow-up, each patient was contacted with regular interval (2 months) by telephone or outpatient review until September 30, 2019, or death, or lost to follow-up.

The primary outcome was overall survival (OS). OS was defined as the time from the date of initial TACE treatment to the date of death or last follow-up (September 30, 2019). The second outcome was progression-free survival (PFS). PFS was defined as the time from the date of initial TACE treatment to the date of radiological progression or death.

### Statistical Analysis

All variables were presented as median [interquartile range (IQR)] for quantitative variables and as count (percentage) for qualitative variables. For continuous variables, the Mann–Whitney *U*-test or Student *t*-test was used. The Fischer’s exact test was used for categorical variables. The body composition features of patients with different Child–Pugh classes and responses (responder and non-responder) were also documented and compared. One-way ANOVA test was used to compare the differences between Child–Pugh classes and tumor responses. Survival curves were calculated using the Kaplan–Meier method and compared with log-rank test. Receiver operating characteristic (ROC) curve and binary logistic regression were performed to evaluate the predictive performance of the body composition features with respect to responder to TACE treatment. The median values of the parameters were considered as cutoff values. The discrimination and performance of body composition features were measured by area under time-dependent ROC curve. Univariate and multivariate Cox proportional hazard analyses were applied to identify the association between body composition parameters and outcomes. Parameters with *p*-value < 0.05 in univariate analysis were included in the multivariate analysis. Variables with *p*-value < 0.05 were regarded statistically significant. All statistical analyses were performed using SPSS 18.0 for Windows (IBM Corporation, Somers, NY, United States) or R version 3.3.2.

## Results

### Patient Characteristics

Among the 192 intermediate stage HCC patients included in the present study, 157 (79.7%) were men and 35 (20.3%) were women. Median body mass index (BMI) was 22.5 (IQR, 20.8–24.2) and median age was 60 (IQR, 52–67). HBV infection (63.5%) was the main etiology, with a median tumor size of 6.3 cm (IQR, 3.0–9.6). There were 179 patients with Child–Pugh A and 13 patients with Child–Pugh B, 98 and 93 patients with ALBI grades 1 and 2, respectively. One hundred five patients of the entire cohort had cirrhosis. The body composition parameters are presented in Table 1. The median value of muscle index, VAT, and SAT were 46.3 (IQR, 39.6–52.8), 38.0 (IQR, 24.6–55.2), and 37.0 (IQR, 27.5–51.1) cm^2^/m^2^, respectively. The median HU of muscle index, VAT, and SAT were 50.2 (IQR, 46.6–54.3), −89.1 (IQR, −96.7 to −77.2), and −103.6 (IQR, −110.7 to −97.2), respectively. For tumor response after the initial TACE response, 129 and 63 patients are responders and non-responders, respectively. The median follow-up was 21.3 months (95% CI, 20.6–22.2). The median OS and PFS of all patients were 20.8 months (95% CI, 18.1–23.7) and 10.6 months (95% CI, 9.2–12.0), respectively.

**TABLE 1:** Baseline demographic and clinical characteristics of patients.

Characteristics	Overall (*n* = 192)
Age	60 (52–67)
Gender (male/female)	157 (81.8%)/35 (18.2%)
BMI	22.5 (20.8-24.2)
Etiology (HBV/Others)	122 (63.5%)/70(36.5%)
Tumor Size (cm)	6.3 (3.0–9.6)
Tumor Number	2 (2–4)
Tumor Location (Unilobar/bilobar)	127 (66.1%)/65 (33.9%)
Cirrhosis (Yes/No)	105 (54.7%)/87 (45.3%)
Ascites (Yes/No)	24 (12.5%)/168 (87.5%)
Child-Pugh Class (A/B)	179 (93.2%)/13 (6.8%)
ALBI grade (1/2/3)	98 (51.0%)/93 (48.5%)/1 (0.5%)
AST (U/L)	44.0 (29.0–61.8)
ALT (U/L)	34.0 (23.4–50.4)
Bilirubin	16.3 (11.6–22.4)
ALB (g/L)	39.8 (36.0–43.9)
AFP (ng/dl)	106.0 (10.1–1000.0)
Muscle index	46.3 (39.6–52.8)
VAT index	38.0 (24.6–55.2)
SAT index	37.0 (27.5–51.1)
Muscle HU	50.2 (46.6–54.3)
VAT HU	−89.1(−96.7 to −77.2)
SAT HU	−103.6(−110.7 to −97.2)
Tumor Response (Responder/non-responder)	129 (67.2%)/63 (32.8%)

*BMI, Body mass index; ALBI, albumin-bilirubin; ALT, alanine transaminase; AST, aspartate transaminase; ALB, albumin; VAT, visceral adipose tissue; SAT, subcutaneous adipose tissue; AFP, alpha-fetoprotein; HU, hounsfield units.*

### Differences in Body Composition Features Among Child–Pugh Classes and Tumor Responses

**FIGURE 1:**
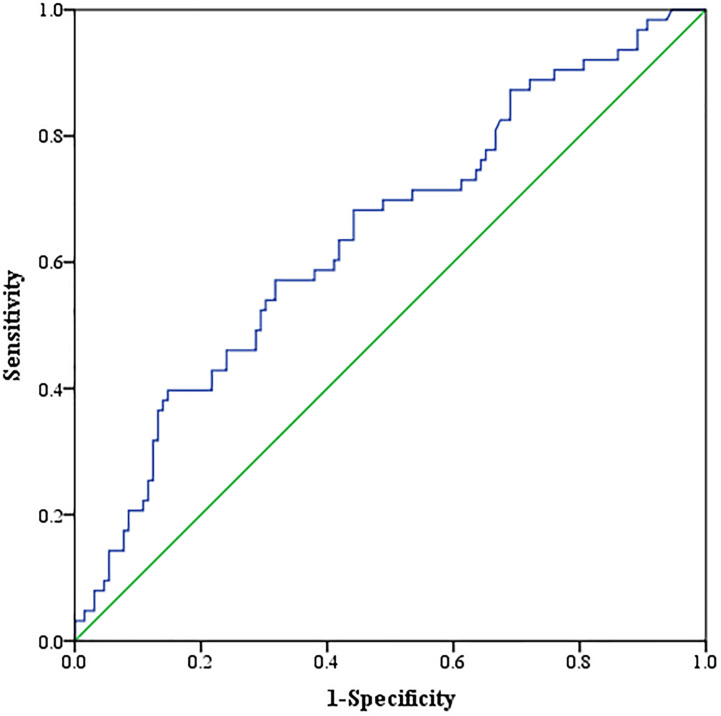
Receiver operating characteristic (ROC) curve analysis concerning the discrimination of visceral adipose tissue (VAT) density between responder and non-responder patients.

Neither muscle mass nor adipose tissue index was identified to be associated with different Child–Pugh classes in the entire cohort (*p* > 0.05) (Supplementary Table 1). For tumor response, we found that a significant variation of VAT HU was detected in the entire group. VAT HU in the responder group tended to be lower than those in the non-responder group [−90.6 (95% CI, −98.2 to −80.7) vs. −81.9 (95% CI, −94.8 to −70.4), *p* = 0.001] (Table 2). In addition, the ROC curve analysis showed that VAT HU was suitable to distinguish between responder and non-responder patients, revealing an AUC value of 0.643 (Figure 1). The VAT density corresponds to the sensitivity values of 57.1% and specificity values of 68.2%, respectively. Moreover, univariate binary logistic regression analysis was applied to further evaluate the association of the VAT density with the tumor response to TACE treatment, showing a statistical significance (odds ratio: 1.035, 95% CI: 1.014–1.058, *p* = 0.001).

**TABLE 2:** Body mass parameters variability across Tumor Response after initial TACE.

Body Mass Parameters	N	Overall	N	CR + PR	N	PD + SD	*P*-value
Muscle index	192	46.3 (39.6–52.8)	129	46.2 (39.5–52.5)	63	46.4 (39.5–53.0)	0.835
Muscle HU	192	50.2 (46.6–54.3)	129	50.6 (46.6–54.3)	63	49.8 (46.8–54.3)	0.548
SAT index	192	37.0 (27.5–51.1)	129	38.7 (27.7–52.4)	63	34.2 (24.8–47.9)	0.133
SAT HU	192	−103.6 (−110.7 to −97.2)	129	−103.9 (−111.1 to −98.5)	63	−102.9 (−109.7 to −95.0)	0.106
VAT index	192	38.0 (24.6–55.2)	129	38.3 (26.1–55.3)	63	34.2 (21.0–53.6)	0.344
VAT HU	192	−89.1 (−96.7 to −77.2)	129	−90.6 (−98.2 to −80.7)	63	−81.9 (−94.8 to −70.4)	0.001
BMI	192	22.5 (20.8–24.2)	129	22.6 (20.8–24.2)	63	22.0 (20.7–24.7)	0.703

*BMI, Body mass index; VAT, visceral adipose tissue; SAT, subcutaneous adipose tissue; HU, hounsfield units.*

### Association of Body Composition Features With Outcomes After Transarterial Chemoembolization

**FIGURE 2:**
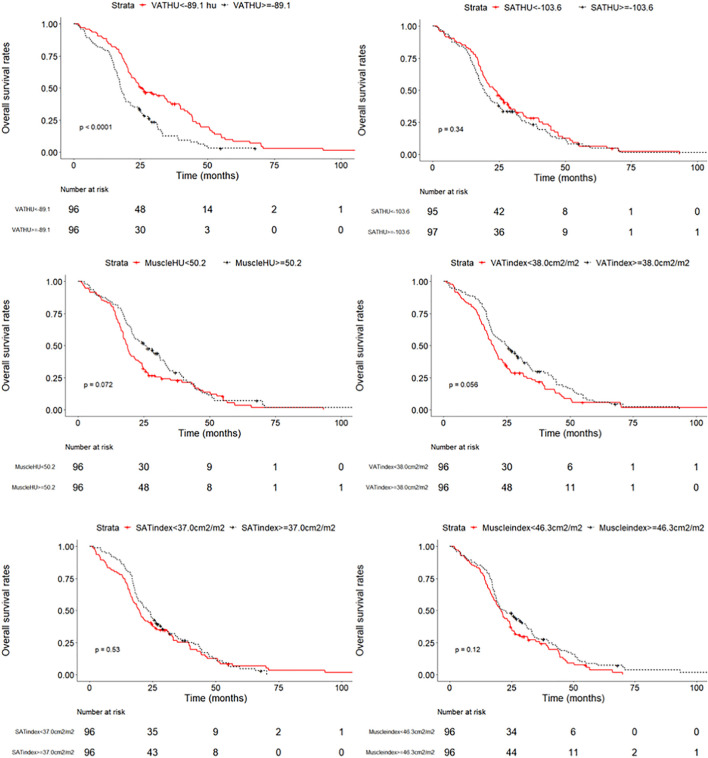
Overall survival curves in patients with intermediate stage hepatocellular carcinoma according to the different body composition parameters.

**FIGURE 3:**
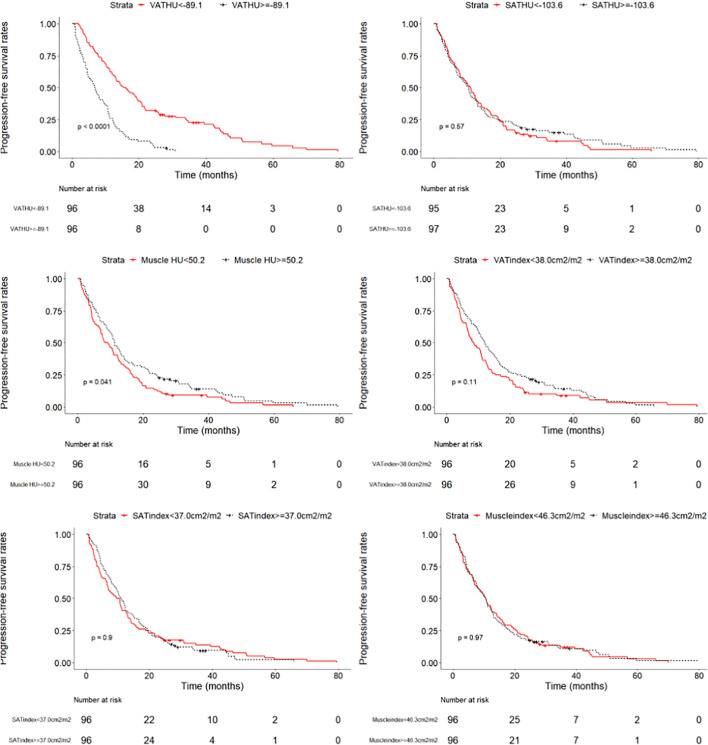
Progression-free survival curves in patients with intermediate stage hepatocellular carcinoma according to the different body composition parameters.

**FIGURE 4:**
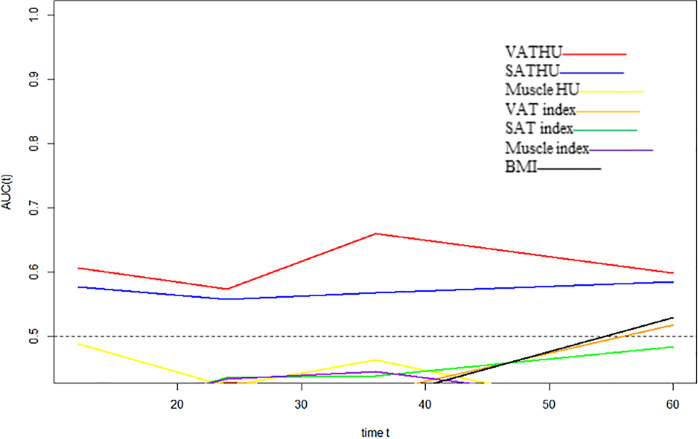
Time-dependent area under the curve (AUC) values of the different body composition parameters in predicting overall survival.

**FIGURE 5:**
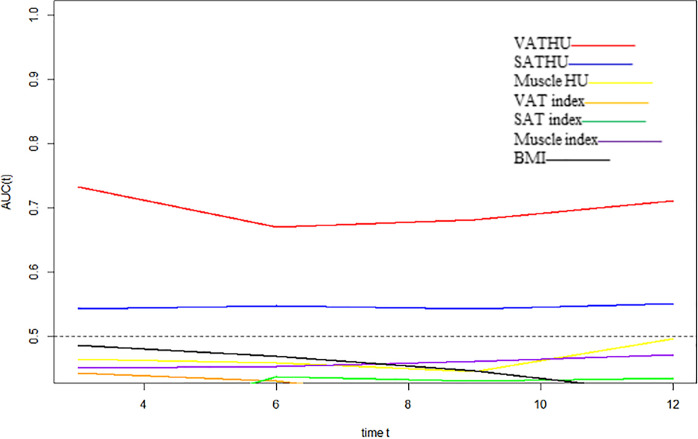
Time-dependent AUC values of the different body composition parameters in predicting progression-free survival.

We evaluated whether the body composition parameters might be associated with the OS and/or PFS. Hence, we divided the entire cohort into two subgroups with respect to their body composition features (either above or below the 50th percentile). By using this cutoff value, a significant prolonged OS and PFS was displayed by Kaplan–Meier curve analysis for HCC patients with VAT HU below −89.1 (25.1 months, 95% CI: 18.1–32.1 vs. 17.6 months, 95% CI: 16.3–18.8, *p* < 0.0001, 15.4 months, 95% CI: 10.6–20.2 vs. 6.6 months, 95% CI: 4.9–8.3, *p* < 0.0001, respectively) (Figures 2, 3). The patients with muscle HU above 50.2 also had a PFS gain (11.3 months, 95% CI: 9.6–13.0 vs. 8.3 months, 95% CI: 5.3–11.3, *p* = 0.041) (Figure 3). The performance and discrimination of the VAT HU and other body composition features were compared (Supplementary Tables 2, 3). The 1-, 2-, 3-, and 5-year OS AUC values of the VAT HU were higher than the other body composition parameters, suggesting a favorable performance and discrimination (Figure 4). Meanwhile, it is also found that 3-, 6-, 9-, and 12-month PFS AUC values of VAT HU were the highest among all the parameters (Figure 5). In order to further investigate the predictive value of the body composition features in the context of TACE treatment, univariate and multivariate Cox-regression analyses were performed with respect to the outcomes, and we detected a significant association between VAT density and outcomes (OS, HR: 1.015, 95% CI: 1.004–1.025, *p* = 0.005, PFS, HR: 1.026, 95% CI: 1.016–1.036, *p* < 0.0001, respectively) (Tables 3, 4).

**TABLE 3:** Univariate and Multivariate analysis of prognostic factors for Overall Survival.

Characteristics	Univariate			Multivariate		
		
	HR	95% CI	*P*-value	HR	95% CI	*P*-value
Muscle index	0.984	0.969–0.998	0.028			0.065
VAT index	0.993	0.986–1.000	0.063			
SAT index	0.993	0.984–1.001	0.103			
Muscle HU	0.989	0.975–1.004	0.139			
VAT HU	1.015	1.004–1.025	0.005	1.015	1.004–1.025	0.005
SAT HU	1.012	1.000–1.024	0.050			0.393
BMI	0.943	0.894–0.995	0.032			0.127

*BMI, Body mass index; VAT, visceral adipose tissue; SAT, subcutaneous adipose tissue; HU, hounsfield units.*

**TABLE 4:** Univariate and Multivariate analysis of prognostic factors for Progression-free Survival.

Characteristics	Univariate			Multivariate		
		
	HR	95% CI	*P*-value	HR	95% CI	*P*-value
Muscle index	0.993	0.980–1.007	0.340			
VAT index	0.992	0.986–0.999	0.020			0.695
SAT index	0.996	0.988–1.004	0.376			
Muscle HU	0.984	0.968–1.001	0.061			
VAT HU	1.026	1.017–1.035	<0.0001	1.026	1.016-1.036	<0.0001
SAT HU	1.007	0.995–1.019	0.233			
BMI	0.959	0.911–1.008	0.102			

*BMI, Body mass index; VAT, visceral adipose tissue; SAT, subcutaneous adipose tissue; HU, hounsfield units.*

## Discussion

The current study showed the prognostic performance of VAT density for intermediate stage HCC patients treated with TACE. We demonstrated that the VAT density was not only associated with survival such as OS and PFS but also tumor response, suggesting that the influence of VAT HU on BCLC B stage HCC patients’ outcomes could reflect tumor-specific factors.

Hepatocellular carcinoma patients with BCLC B stage are a group of highly heterogenous in terms of variable liver function, tumor burden, and disease etiology, leading to various individual responses (Arizumi et al., 2015). Furthermore, repeated TACE may induce impaired liver function and has an impact on the prognosis of the inter-mediated stage HCC patients (Kudo et al., 2014). To prolong OS of the intermediate-stage HCC patients, it is crucial to identify the target population who may benefit from TACE and make risk stratification. There remains considerable uncertainty, which the existing guidelines do not adequately address criteria for prognostic factors, given the diversity of clinical responses (Vitale et al., 2018). Except the well-knowledged factors such as alpha-fetoprotein, bilirubin, performance status, the other non-invasive and simply applied factors are still on the prowl.

In recent years, previous evidence suggested that there is a correlation between obesity and prognosis of malignancies including HCC (Charette et al., 2019). In addition, non-alcoholic fatty liver disease (NAFLD), which is becoming the first cause of chronic liver disease, is the risk factor of HCC development and prognosis of HCC patients (Seror et al., 2021). Although the body mass index (BMI) is widely applied to characterize the body compositions, limitations persist in its use such as inability to differentiate fat and muscle mass (Strulov Shachar and Williams, 2017). In contrast, analytic morphomics, using semi-automated image-processing platform to evaluate body composition, was considered as offering an accurate approach to quantitate not only the aggressive nature of HCC itself but the underlying HCC patients’ characteristics such as liver status (Singal et al., 2016). Moreover, with its available resolution of adipose tissue, CT scan is the gold standard quantitative assessment of tissue density (Doyle et al., 2013).

Previous studies showed that both SAT and VAT density were negatively correlated with survival, suggesting that an increased adipose tissue attenuation could be used as a novel non-invasive biomarker for predicting prognosis (Murphy et al., 2014; Rosenquist et al., 2015). More recently, Hessen et al. suggested that high SAT density correlates negatively with OS in patients with HCC (von Hessen et al., 2021). Our study showed that VAT density was significantly in correlation with tumor response (CR + PR) (odds ratio: 1.035, 95% CI: 1.014–1.058, *p* = 0.001). As such, a pre-TACE VAT HU below −89.1 was an independent predictor for favorable outcomes. HCC patients in this cohort below the cutoff value presented a median OS and PFS of 25.1 and 15.4 months compared with 17.6 and 6.6 months in patients with a VAT HU above the cutoff, highlighting the value of VAT in predicting outcomes of inter-mediated stage HCC patients undergoing TACE. Iwase et al. (2020) reported that a high VAT area was correlated with a reduced disease-free survival in breast cancer patients undergoing neoadjuvant chemotherapy. In general, SAT and VAT are two main compartments with different metabolic characteristics of body fat tissue (Shuster et al., 2012). Compared with SAT, previous studies showed the association between VAT and various pathologies such as insulin resistance, impaired glucose, and lipid metabolism could relate to the prognosis of patients with cancers, and VAT was regarded to be more pro-tumorigenic and pro-inflammatory (Neeland et al., 2019; Li et al., 2020). Moreover, hormones and bioactive molecules including interleutin 6 (IL-6), tumor necrosis factor, adiponectin, and resistin are released by the VAT (Shuster et al., 2012). Insulin can irritate the proliferation of HCC cells and accelerate the vascular invasion of HCC (Karagozian et al., 2014). In particular, considering that adiponectin has protective antiangiogenic activity, regulating the vascular endothelial growth factor (VEGF) levels induced by TACE could have an impact on outcomes of HCC patients (Bagchi et al., 2013). The reasons for only high VAT density had a negative impact on outcomes are speculated as follows: First, adipose tissue density might be qualitative biomarker and a high adipose tissue density is in correlation with a depletion of adipose, which could reflect the poor nutritional condition (Charette et al., 2019). In addition, the underlying diseases in HCC patients and chronic inflammation could also lead to a higher VAT density (Batista et al., 2016). Finally, in patients with cancer cachexia, the high CT density of fat tissue, which is determined by fat and lipid could result from the activation of brown adipose tissue (Beijer et al., 2012). Interestingly, a cutoff value of −89.1 HU, which has been demonstrated in the present study, was close to the previous study in HCC patients, with a cutoff value of −88 HU (von Hessen et al., 2021).

Numerous studies showed that sarcopenia, which is defined as a progressive and generalized skeletal disorder, was considered associated with a higher incidence of adverse events and poor prognosis in HCC patients treated with various therapies (Choi et al., 2020; Uojima et al., 2020). Nevertheless, there is little data concerning the prognosis value of sarcopenia in HCC patients receiving TACE (Marasco et al., 2020). This study suggested that patients with a higher muscle density had a PFS gain, whereas the muscle mass and density had no correlation with the OS and tumor response. Kobayashi et al. (2018) and Fujita et al. (2019) showed there was no significance between pre-TACE muscle mass and clinical outcomes. In contrast, two other studies indicated pre-TACE sarcopenia was an independent factor of negative outcomes (Dodson et al., 2013; Loosen et al., 2019). These finding demonstrated that the role of the sarcopenia in predicting prognosis may mainly depend on the general clinical status of HCC patients and not directly on the local response of TACE treatment (Marasco et al., 2020).

Of note, there are some limitations in this study. First, it is a small sample retrospective study and subject to collection and selection bias. HCC patients with unavailable CT scans were excluded in this study. Additionally, the lack of a control group represents limit in the convincing evidence of the analysis. The results also need to be confirmed with external validation. Finally, changes in the body composition parameters after TACE treatment were not analyzed in the study. The impact of changes of these parameters on the outcomes of intermediate stage HCC patients treated with TACE should be further conducted with future large sample size studies.

In conclusion, the VAT density could provide prognostic prediction value and may be helpful to stratify the BCLC B stage patients in order to optimize the selection criteria for undergoing TACE treatment.

## Data Availability Statement

The raw data supporting the conclusions of this article will be made available by the authors, without undue reservation.

## Ethics Statement

The current study was reviewed and approved by the Institutional Review Board of the Soochow University and was conducted in accordance with the ethical standards laid down in the Declaration of Helsinki. The requirement for informed consent was waived due to its retrospective nature.

## Author Contributions

All authors contributed to the review and critical revision of the manuscript and approved the final version of the manuscript. TC, C-FN, QL, and LZ contributed to the study concept and design. QL, LZ, Z-HH, D-XZ, and SZ contributed to the acquisition of data. LZ, Z-HH, D-XZ, and J-BL contributed to the analysis and interpretation of the data. LZ, Z-HH, D-XZ, and YY contributed to the statistical analysis. QL, LZ, Z-HH, and D-XZ contributed to the drafting of the manuscript. The corresponding author had full access to all the data and took full responsibility for the veracity of the data and the statistical analyses.

## Conflict of Interest

The authors declare that the research was conducted in the absence of any commercial or financial relationships that could be construed as a potential conflict of interest.

## Publisher’s Note

All claims expressed in this article are solely those of the authors and do not necessarily represent those of their affiliated organizations, or those of the publisher, the editors and the reviewers. Any product that may be evaluated in this article, or claim that may be made by its manufacturer, is not guaranteed or endorsed by the publisher.

## Abbreviations

HCC: hepatocellular carcinoma
BCLC: Barcelona Clinic Liver Cancer
TACE: transarterial chemoembolization
OS: overall survival
PFS: progression-free survival
IRBs: Institutional Review Boards
CT: computed tomography
BMI: body mass index
HU: Hounsfield units
VEGF: vascular endothelial growth factor
MRI: magnetic resonance imaging
SM: skeletal muscle
VAT: visceral adipose tissue
SAT: subcutaneous adipose tissue
HR: hazard ratio
IQR: interquartile range
mRECIST: modified Response Evaluation Criteria in Solid Tumors
CI: confidence interval.
