# Supplementary material for: High Visceral Adipose Tissue Density Correlates With Unfavorable Outcomes in Patients With Intermediate-Stage Hepatocellular Carcinoma Undergoing Transarterial Chemoembolization
Source: Front Cell Dev Biol. 2021 Sep 8;9:710104. doi: 10.3389/fcell.2021.710104 (PMC8455878; doi:10.3389/fcell.2021.710104)
Supplement: Supplementary file 1 [file Data_Sheet_1.DOCX]

Table S1: Body mass parameters variability across Child Pugh Classes

| Body mass parameters | N | Overall | N | Child Pugh Class A | N | Child Pugh Class B | P value |
| --- | --- | --- | --- | --- | --- | --- | --- |
| Muscle index | 192 | 46.3(39.6-52.8) | 179 | 46.6(39.4-52.8) | 13 | 44.3(40.5-52.5) | 0.234 |
| Muscle HU | 192 | 50.2(46.6-54.3) | 179 | 50.3(46.8-54.4) | 13 | 49.1(43.6-53.1) | 0.655 |
| SAT index | 192 | 37.0(27.5-51.1) | 179 | 36.0(27.5-51.0) | 13 | 43.8(31.1-58.8) | 0.838 |
| SAT HU | 192 | -103.6(-110.7- -97.2) | 179 | -103.8(-110.7--97.3) | 13 | -102.0(-110.1--93.9) | 0.177 |
| VAT index | 192 | 38.0(24.6-55.2) | 179 | 37.9(24.3-55.3) | 13 | 38.2(31.7-52.9) | 0.428 |
| VAT HU | 192 | -89.1(-96.7--77.2) | 179 | -89.3(-97.1--77.0) | 13 | -85.6(-94.4--77.1) | 0.432 |
| BMI | 192 | 22.5(20.8-24.2) | 179 | 22.5(20.8-24.2) | 13 | 22.0(20.1-24.1) | 0.415 |

BMI, Body mass index; VAT, visceral adipose tissue; SAT, subcutaneous adipose tissue; HU, hounsfield units

Table S2 Comparison of the performance and discriminative ability of body composition features in predicting overall survival.

|  | 1. yr AUROC   (95%CI) | 1. yr AUROC   (95%CI) | 1. yr AUROC   (95%CI) | 5-yr AUROC  (95%CI) | LR χ^2^ | 1. Index (95%CI) | Df |
| --- | --- | --- | --- | --- | --- | --- | --- |
| VATHU | 0.607(0.499-0.714) | 0.573(0.490-0.656) | 0.661(0.562-0.759) | 0.599(0.446-0.751) | 7.51 | 0.580(0.531-0.629) | 1 |
| SATHU | 0.577(0.459-0.695) | 0.558(0.476-0.640) | 0.568(0.468-0.668) | 0.586(0.425-0.747) | 3.58 | 0.550(0.499-0.601) | 1 |
| Muscle HU | 0.488(0.370-0.607) | 0.424(0.342-0.505) | 0.464(0.364-0.564) | 0.368(0.139-0.596) | 1.74 | 0.549(0.496-0.602) | 1 |
| VAT Index | 0.379(0.269-0.489) | 0.429(0.347-0.512) | 0.413(0.310-0.515) | 0.518(0.341-0.696) | 3.66 | 0.554(0.503-0.605) | 1 |
| SAT Index | 0.368(0.264-0.472) | 0.437(0.354-0.519) | 0.439(0.336-0.541) | 0.448(0.269-0.627) | 2.75 | 0.558(0.509-0.6070 | 1 |
| Muscle Index | 0.377(0.268-0.485) | 0.434(0.352-0.516) | 0.446(0.337-0.555) | 0.388(0.205-0.571) | 4.85 | 0.560(0.509-0.611) | 1 |
| BMI | 0.342(0.238-0.445) | 0.409(0.327-0.491) | 0.403(0.296-0.510) | 0.530(0.327-0.733) | 4.74 | 0.572(0.521-0.623) | 1 |

BMI, Body mass index; VAT, visceral adipose tissue; SAT, subcutaneous adipose tissue; HU, hounsfield units

Table S3 Comparison of the performance and discriminative ability of body composition features in prediction progression-free survival.

| Parameters | 3-month AUROC  (95%CI) | 6-month AUROC  (95%CI) | 1. month AUROC   (95%CI) | 12-month AUROC  (95%CI) | LR χ^2^ | C-Index  (95%CI) | Df |
| --- | --- | --- | --- | --- | --- | --- | --- |
| VATHU | 0.733(0.648-0.818) | 0.671(0.592-0.749) | 0.681(0.606-0.757) | 0.711(0.637-0.785) | 27.65 | 0.641(0.602-0.680) | 1 |
| SATHU | 0.543(0.427-0.659) | 0.548(0.462-0.634) | 0.544(0.461-0.627) | 0.551(0.469-0.633) | 1.36 | 0.531(0.484-0.578) | 1 |
| Muscle HU | 0.465(0.341-0.589) | 0.460(0.371-0.548) | 0.446(0.364-0.529) | 0.497(0.415-0.580) | 2.71 | 0.526(0.479-0.573) | 1 |
| VAT Index | 0.444(0.339-0.549) | 0.430(0.345-0.516) | 0.396(0.317-0.476) | 0.387(0.306-0.468) | 5.83 | 0.564(0.517-0.611) | 1 |
| SAT Index | 0.353(0.249-0.457) | 0.438(0.349-0.526) | 0.431(0.349-0.513) | 0.435(0.354-0.517) | 0.81 | 0.543(0.498-0.588) | 1 |
| Muscle Index | 0.452(0.337-0.566) | 0.453(0.366-0.541) | 0.462(0.380-0.544) | 0.472(0.389-0.554) | 0.91 | 0.524(0.475-0.573) | 1 |
| BMI | 0.487(0.368-0.606) | 0.470(0.382-0.557) | 0.446(0.364-0.528) | 0.412(0.330-0.493) | 2.76 | 0.554(0.505-0.603) | 1 |

BMI, Body mass index; VAT, visceral adipose tissue; SAT, subcutaneous adipose tissue; HU, hounsfield units
